# The Cross-Knit Between Immune Cells and Thyroid Function in Autoimmune Thyroid Disorders: What We Can Learn from Inborn Errors of Immunity

**DOI:** 10.3390/children13020169

**Published:** 2026-01-25

**Authors:** Laura Grilli, Francesca Cillo, Roberta Romano, Giuliana Giardino, Francesca Romana Rotondo, Sara Vasaturo, Mariacarolina Salerno, Donatella Capalbo

**Affiliations:** 1 Pediatric Immunology Unit, Department of Translational Medical Sciences, University of Naples Federico II, 80138 Naples, Italy; 2 Department of Mother and Child, University Hospital Federico II, ERN-RITA Center for Rare Immundeficiencies, 80138 Naples, Italy; 3 Department of Mother and Child, University Hospital Federico II, Endo-ERN Center for Rare Endocrine Conditions, 80138 Naples, Italy; 4 Pediatric Endocrinology Unit, Department of Translational Medical Sciences, University of Naples Federico II, 80138 Naples, Italy

**Keywords:** autoimmune thyroid diseases, immunodysregulation, inborn errors of immunity

## Abstract

**Highlights:**

**What are the main findings?**

- Autoimmune thyroid diseases (AITDs) share key pathogenetic mechanisms with inborn errors of immunity (IEIs), making IEIs a natural model to study immune tolerance breakdown.
- Genetic and epigenetic perturbations affecting central and peripheral tolerance mechanisms contribute to the development of thyroid autoimmunity.

**What is the implication of the main findings?**

- Understanding how specific immune defects lead to AITDs enables the identification of precise molecular pathways driving thyroid autoimmunity.
- These insights support the development of targeted, pathway-specific therapies and precision-medicine approaches for AITDs.

**Abstract:**

Autoimmune thyroid diseases (AITDs), including Hashimoto thyroiditis and Graves’ disease, are the most common autoimmune endocrinopathies, affecting up to 5% of the population. Pathogenetic pathways have not yet been fully elucidated, even though different immune-genetic alterations have been proposed. Specific immune defects presenting with AITDs may serve as an experimentum naturae to study the involvement of a specific pathway in the pathogenesis of the disease. In fact, since immune dysregulation with autoimmunity frequently characterize inborn errors of immunity (IEIs), understanding the mechanisms of immune tolerance breakdown leading to autoimmunity in these conditions may provide useful insight to understand the pathogenesis of AITDs. In this review, we will highlight the main immunological aspects of AITDs and their pathogenesis in IEIs.

## 1. Introduction

Autoimmune thyroid diseases (AITDs) encompass a spectrum of disorders mainly represented by Graves’ disease (GD) and Hashimoto’s thyroiditis (HT). These conditions represent the most prevalent autoimmune endocrinopathies, affecting up to 5% of the general population [1]. GD and HT, although sharing underlying autoimmune mechanisms, are characterized by distinct clinical presentations and pathophysiological profiles [2].

GD is the leading cause of hyperthyroidism in iodine-sufficient regions and it is characterized by the presence of TSH receptor autoantibodies (TRAb), which chronically stimulate the thyroid gland, resulting in diffuse goiter, elevated thyroid hormone levels, and clinical signs such as weight loss, palpitations, and heat intolerance. A notable feature of GD is the possible involvement of orbital tissues, leading to Graves’ orbitopathy, a potentially severe extrathyroidal manifestation [3,4]. In contrast, HT is the most common cause of hypothyroidism and it is characterized by progressive lymphocytic infiltration of the thyroid gland, leading to thyroid follicles destruction, decreased hormone production, and eventual gland atrophy. It is typically associated with high titers of anti-thyroid peroxidase (TPO-Ab) and anti-thyroglobulin (Tg-Ab) antibodies. Patients often present with fatigue, weight gain, cold intolerance, and a firm, painless goiter [2,5,6]. Diagnosis of both AITDs involves a combination of clinical evaluation, thyroid function tests (TSH, free T4), and serological markers specific to each condition, such as TRAb for GD and TPO-Ab/Tg-Ab for HT. HT is the most common cause of acquired hypothyroidism in children. Early diagnosis of thyroid disorders is crucial. In particular, thyroid function affects neurodevelopment in the pediatric population potentially leading to long-term consequences. Thyroid dysfunction may also influence metabolic processes, which could in turn be associated with an increased cardiovascular risk [7,8,9]. The role of thyroid imaging, especially ultrasound, is pivotal in the diagnostic workup. In HT, ultrasound typically reveals a hypoechoic, heterogeneous glandular texture, sometimes with pseudonodular patterns, while in GD, it shows increased vascularity (“thyroid inferno”) and a diffusely enlarged gland [10]. Despite the well-described clinical and serological profiles, the immunopathogenesis of AITDs remains incompletely understood. Current evidence suggests that a multifactorial interplay of genetic predisposition, environmental triggers, and epigenetic modifications contributes to the initiation and progression of the autoimmune response, ultimately leading to thyroid tissue destruction [5]. Recent advances highlight the role of impaired regulatory T-cell function, cytokine-mediated apoptosis of thyrocytes, and defects in immune tolerance as central mechanisms in disease development [11]. From a therapeutic perspective, the standard treatment of GD includes antithyroid drugs (primary methimazole), radioiodine therapy, or thyroidectomy, depending on disease severity, recurrence, and patient preference [12,13]. For HT, therapy primarily involves levothyroxine replacement, especially in overt hypothyroidism [5]. Emerging therapeutic strategies are currently under investigation to improve long-term management and minimize relapses. Advances in understanding the immune and molecular mechanisms of AITDs are opening new avenues for more personalized and targeted interventions, beyond symptomatic hormone control [12].

## 2. Autoimmune Thyroid Diseases and Immune Tolerance Breakdown

Different autoimmune diseases, including AITDs, share common pathogenetic mechanisms in which immune system dysregulation, resulting from the interaction between genetic susceptibility and environmental factors, leads to an organ specific immune attack. Although the underlying mechanisms are not yet fully understood, numerous studies have focused on elucidating the role of regulatory T cells (Tregs), which are essential for maintaining immune homeostasis through their potent suppressive functions. A reduction in Tregs has been reported in patients with HT, particularly in the activated regulatory T cells subpopulation (CD45RO^+^ Foxp3 ^high^), which exhibits strong suppressive activity in vivo. Moreover, the frequency of these cells has been shown to undergo dynamic changes during the progression of the disease [14,15]. Moreover, the PD-1/PD-L1 pathway, a key regulator of peripheral immune tolerance, has been shown to be dysregulated in AITDs. In particular, PD-L1 expression has been detected in thyroid follicular cells and appears to be induced by multiple factors, including IFN-γ or the loss of close contact with neighboring thyroid follicular cells (TFCs). This pathway seems to contribute to limiting the autoimmune response and to slowing disease progression; however, its activity is not sufficient to achieve complete immune suppression. Based on these observations, it can be hypothesized that enhancing the expression of inhibitory immune checkpoint receptors on target cells may confer protection against autoimmunity [15,16]. The environmental triggers implicated in the breakdown of immune tolerance are only partially known [17,18]. Among the recognized triggers, infections play a key role through different pathogenetic mechanisms including molecular mimicry, bystander activation, epitope spreading, and exposure of cryptic antigens [19,20]. Molecular mimicry consists of the similarity in terms of structure and/or sequence between self and infectious agents’ antigens, resulting in a cross-reactive immune response [20,21]. The inflammatory milieu associated with infectious diseases can trigger the nonspecific mechanism of bystander-activation, clones of autoreactive B and T cells [22]. Moreover, prolonged tissue damage can unmask cryptic intracellular antigens with new epitope spreading [23]. The hypothesis of the implication of infections as a trigger of AITDs is supported by evidence showing its association with bacterial infectious agents such as Yersinia enterocolitica, Borrelia burgdorferi and Helicobacter pylori [24,25]. Also viruses, including more recently SARS-CoV-2, have been identified as a major environmental factor involved in AITDs [26,27]. ACE2 expression, crucial for the viral recognition and subsequent fusion to cell membrane, was observed to be upregulated within thyroid tissues in patients affected with COVID-19. The thyroid gland can be directly infected by SARS-CoV-2 as the ACE2 and other factors facilitating viral entry are highly expressed in it. On the other hand, the gland is indirectly affected when SARS-CoV-2 infection triggers the cytokine storm [28].

Patients with AITDs are prone to develop other autoimmune comorbidities. In previous studies an association between AITDs, celiac disease (CD) and type 1 Diabetes Mellitus (DM1) strongly emerged. Currently, the coordinated screening of these comorbid autoimmune conditions is indicated at the time of diagnosis and periodically during the follow-up [29,30].

AITDs are the most frequent endocrinopathies, occurring in 14–25% of children with DM1 [31,32]. However, even though a great proportion of these patients show thyroid autoantibodies (Ab anti-thyroperoxidase [TPO] and Ab anti-tireoglobulin [TG]), only a few of them develop clinical hypothyroidism in childhood and adolescence. Indeed, in most cases the patients maintain a normal thyroid function and, in some cases, they progress towards subclinical hypothyroidism [33]. Similarly, CD is significantly more common in AITDs patients [34] and on the other hand in pediatric patients with CD a higher prevalence of thyroid autoimmunity compared to the general population is reported [35]. Two underlying hypotheses have been proposed for these associations: a common genetic substrate as HLA alleles and continued gluten exposure leading to the intestinal barrier damage and induction of systemic immune response [36].

However, in a large multicenter Italian study, no difference of AITDs prevalence between gluten free diet and untreated CD patients has been observed [37].

### Thyroid Specific Mechanism of Immune Dysregulation

Beyond systemic immune tolerance breakdown, autoimmune thyroid diseases are characterized by tissue-specific mechanisms that make the thyroid a preferential target of autoimmune attack. Under inflammatory conditions, thyrocytes upregulate HLA class I and class II molecules and express costimulatory and adhesion molecules such as CD40 and ICAM-1, enabling them to present thyroid-specific antigens such as TG, TPO and TSHR directly to autoreactive T cells, amplifying local immune responses [16]. The iodination of TG, an essential step in thyroid hormone synthesis, increases its immunogenicity and facilitates epitope spreading, thereby enhancing the activation of autoreactive T and B cells. Similarly, TPO and TSHR display conformational epitopes that are preferentially targeted by pathogenic autoantibodies, contributing to either destructive thyroiditis or functional stimulation, as observed respectively in HT and GD. Pro-inflammatory cytokines such as IFN-γ and TNF-α, produced by infiltrating Th1 lymphocytes, further amplify this process by promoting thyrocyte dysfunction and apoptosis, leading to follicular destruction and gland failure in HT [16,38]. Moreover, the biochemical environment of the thyroid gland further contributes to its vulnerability. Thyroid hormone synthesis requires continuous production of hydrogen peroxide, exposing thyrocytes to high oxidative stress. In the presence of chronic inflammation, reactive oxygen species promote post-translational modifications of thyroid antigens and enhance their immunogenicity, reinforcing local immune responses. Excess iodine intake exacerbates this process by increasing antigen iodination and by skewing the balance between effector T cells and regulatory T cells within the thyroid microenvironment. The combined effects of impaired immune tolerance and intrinsic thyroid-specific susceptibility explain the preferential involvement of the thyroid gland in autoimmune disorders.

## 3. Genetic Susceptibility

Many epidemiological investigations, in particular family and twins studies support a strong genetic influence on AITD etiology [18].

Approximately 50% of the siblings of GD patients have thyroid antibodies and up to 33% of those with AITD share the diagnosis with their siblings [39]. Twin studies are strongly convincing, since they show higher concordance for AITD between monozygotic twins than dizygotic twins and have suggested that the overall heritable, genetic contribution to the development of GD is about 75% [40]. AITD susceptibility genes can be categorized as thyroid specific or immune-modulating [2]. Several Single Nucleotide Polymorphisms (SNPs) within the *TG* gene can independently influence AITD disease risk [41,42]. Intron 1 SNPs in the *THSR* gene may affect gene expression/splicing, possibly in the thymus and have been associated with GD predisposition. As expected, the immune-genetic substrate of AITD is shared with other autoimmune conditions. Early studies showed association of HLA-DR 3 with GD [43]; more specifically, HLA-DRβ1-Arg74 has been found to play a key role in the development of AITD, since it induces a conformational change in the HLA-DR molecule with a more positively charged peptide pocket, that potentially eases the presentation of thyroid peptides [44]. In addition, HLA-DR3 is involved in the predisposition to HT [45].

CD40 signaling cascade has been also shown to play a role in several autoimmune conditions [46]. CD40 has been identified as a susceptibility gene for GD, but not for HT. Since GD is a classical antibody mediated disease, it is predictable that B cells would play a key role in its pathogenesis [47]. In fact, Jacobson et al. in 2005, demonstrated that a SNP located in the Kozak sequence of the *CD40* gene, which is critical for the start of translation, leads to the increase of the amount of CD40 on B cells surface [48]. B cells expressing more CD40 have a lower threshold for activation. This leads to over stimulation of the CD40 pathway that in turn elicitates an autoimmune response. In addition, it has been demonstrated that CD40 is expressed on thyrocytes [49] and its levels are increased in GD [50]. Several CTLA-4 SNPs have been associated with an increased risk of AITDs, both GD and HT [51,52]. Since CTLA-4 acts as a major negative modulator of T cell-mediated immune functions, this gene was found to be involved in a variety of autoimmune conditions [53]. Furthermore, according to some authors, CTLA-4 may influence the severity of the AITD phenotype [54,55].

## 4. Epigenetic Factors and ECDs

Epigenetic mechanisms are increasingly recognized as pivotal contributors to the pathogenesis of HT. In particular, alterations in DNA methylation patterns and the deregulated expression of non-coding RNAs have emerged as key factors in modulating immune tolerance and thyroid autoimmunity [5]. Genome-wide and targeted DNA methylation studies have highlighted DNA methylation as a fundamental epigenetic factor in the pathogenesis of AITD. Aberrant methylation can silence tolerance-related genes or activate pro-inflammatory pathways, facilitating lymphocytic infiltration of the thyroid [56]. Moreover, distinct methylation patterns observed between GD and HT suggest that epigenetic differences may contribute to the mechanisms determining disease phenotype, adding further complexity to AITD etiology [57]. Non-coding RNAs, particularly microRNAs (miRNAs), play a central role in fine-tuning immune responses and thyrocyte survival. For instance, miR-146a is consistently upregulated in AITD patients, where it modulates the TLR4/NF-κB pathway through TRAF6, thereby influencing inflammatory signaling despite its expected role in limiting Th1 differentiation. Similarly, miR-142 isoforms are overexpressed, promoting CD4+ T cell differentiation toward pathogenic Th1 phenotypes, impairing Treg homeostasis, and downregulating the tight junction protein CLDN1, which increases thyrocyte permeability to autoantibodies. In addition, miR-301a is elevated in HT and sustains NF-κB activation by inhibiting NKRF, contributing to Th17 cell generation and chronic immune activation [58,59]. Collectively, these findings underscore that epigenetic modifications, and particularly miRNA-mediated regulation, represent a key interface between genetic susceptibility and environmental triggers in AITD, offering promising avenues for biomarker discovery and targeted therapeutic strategies [60]. Furthermore, growing evidence indicates that also environmental endocrine-disrupting chemicals (EDCs) may represent significant triggers of AITDs, partly mediated by epigenetic alterations affecting immune and thyroid-related genes [61]. Beyond their direct thyroid-disrupting properties, EDCs are increasingly recognized as immunomodulatory agents. Experimental and epidemiological studies suggest that these compounds also promote oxidative stress, cytokine imbalance, and loss of immune tolerance, thereby facilitating autoimmune responses [62,63].

Compounds such as per- and polyfluoroalkyl substances (PFAS), bisphenols, and phthalates can interfere with multiple checkpoints of the hypothalamic–pituitary–thyroid axis, including thyroid hormone synthesis, transport, metabolism, and receptor-mediated action, with measurable effects on TSH and circulating thyroid hormones, particularly during critical windows of susceptibility such as fetal life and early childhood [61,63,64]. Furthermore, a recent systematic review reported a positive association between exposure to major EDC classes including bisphenols, phthalates, and PFAS and the risk of autoimmune disorders, supporting the hypothesis that immune disruption may synergize with thyroid-specific toxicity [62].

Taken together, these data support a multifactorial model in which EDCs may contribute to the development of thyroid autoimmunity in genetically susceptible individuals. However, further evidence is required to clarify the specificity of these effects.

## 5. Inborn Errors of Immunity and Thyroiditis

**Figure 1:**
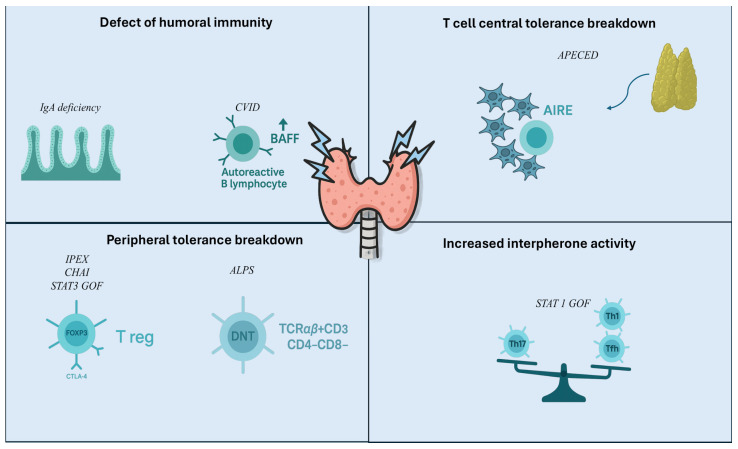
Immune tolerance breakdown in AITD.

Immune dysregulatory manifestations, including autoimmunity, often characterize IEIs, being observed in about 25% of the patients with IEIs, also as predominant phenotype. (Figure 1) [19,65,66] AITDs represent one of the possible expression of organ specific autoimmune response and together with autoimmune cytopenias, entheropathy and skin diseases they are among the most frequent autoimmune clinical conditions in IEIs [67]. IEIs constitute a unique model to investigate the pathogenesis of autoimmune conditions, since several different mechanisms may be involved at the same time in the same form of IEIs. In IEIs autoimmunity may result from a loss of self-tolerance or from the inability of the immune system to eliminate pathogens, which leads to prolonged and exaggerated inflammatory response [68]. Thus, in this paragraph we will summarize the mechanisms of immune tolerance breakdown in IEIs leading to autoimmune diseases, focusing in particular on those associated with AITDs, and we will discuss the implication of the specific gene defects implicated. This approach could lead to the definition of candidate specific targets for the development of specific treatments, such as “precision medicine”, in order to minimize side effects or even preventing autoimmune conditions [69]. IEIs associated with AITDs are described in Table 1.

### 5.1. Defects of Humoral Immunity

#### 5.1.1. Selective IgA Deficiency (SIgAD)

Selective IgA deficiency (SIgAD), defined as totally or partially decreased IgA levels in presence of normal level of other isotypes, is the most common IEI. It is usually considered as a mild condition due to accidental diagnosis and asymptomatic or mildly symptomatic course and the phenotypic manifestations are variable among different patients [70,71]. As a result of IgA importance in mucosal immunity homeostasis, clinical implications include increased risk of sinopulmonary infections with bacteria and viruses and gastrointestinal infections, in particular Giardia lamblia [71,72,73]. Among non-infectious manifestations there are autoimmunity, atopy and malignancy. In particular, immune dysregulation is a common feature in IgA deficient patients including AITDs, DM1 and CD. The mechanisms leading to immune dysregulation in SIgAD have not been fully clarified and it seems to follow multiple ways. On one side, the lower mucosal response causes an increased risk of infectious events and consequently chronic inflammation, correlated with epitope spreading, molecular mimicry and bystander activation [20,74]. On the other side, SIgAD shares the same HLA haplotype of susceptibility (HLA-A1, B8, DR3 and DQ2) associated to several organ specific diseases, including AITDs [75,76]. Moreover some studies also describe a direct role of IgA and IgA receptor (ITAM-bearing receptor) in the regulation of immune response, enhancing and suppressing inflammation [77,78].

#### 5.1.2. Common Variable Immunodeficiency (CVID)

Common Variable Immunodeficiency (CVID) is the most prevalent symptomatic immunodeficiency and includes a group of disorders characterized by reduced serum Ig levels and reduced antibody response to pathogens and vaccines. The main clinical features are a high susceptibility to infections, autoimmunity and an elevated risk of malignancies [79]. Autoimmune conditions occur in approximately 20–30% of patients, with cytopenia being the most frequent [80]. CVID patients often suffer from hypothyroidism, which is the most frequent organ specific autoimmune condition, occurring in up to 3.55 of patients (3.5%) [81]. GD has also been described [72]. The genetic substrate of the disease and general pathological mechanisms leading to autoimmunity remain unclear. However, high levels of BAFF cytokine, promoting B cells maturation and survival and inhibiting the negative selection of autoreactive clones, were found in CVID [82]. Elevated BAFF levels seem to contribute to the increased prevalence of autoimmunity observed in patients with BAFF receptor TACI (transmembrane activator and calcium-modulator and cyclophilin ligand interactor) mutations. TACI mutations account for 10% of genetic variants described in CVID patients [83]. This receptor is supposed to be involved in B cell central tolerance and its reduced function determines a loss of tolerance and thus autoimmunity [84].

### 5.2. IEIs with Disruption of T-Cell Central Tolerance

#### APECED (Autoimmune Polyendocrine—Candidiasis—Ectodermal—Distrophy)

APECED (Autoimmune polyendocrine—Candidiasis—Ectodermal—Distrophy), also known as Autoimmune Polyendocrine Syndrome type 1 (APS1), is a rare autosomal recessive disorder, characterized by a variable combination of chronic mucocutaneous candidiasis (CMC), polyendocrinopathy and dental and nail dystrophy [68]. As the triad of CMC, hypoparathyroidism, and adrenal failure constitutes the most typical presentation of the disease, it should be suspected when at least two clinical features are present [85]. Although this triad represents the classic presentation, numerous atypical variants have been reported, showing considerable variability even within the same family, suggesting the absence of a strict genotype–phenotype correlation [86]. The clinical spectrum of APS-1 may include isolated, rare, or unusual autoimmune or immune-mediated features that can appear years before the hallmark components of the syndrome. These early signs may involve atypical expressions of polyendocrinopathy—thyroiditis being one of the most frequent—or even uncommon dermatological manifestations [87,88].

The spectrum of manifestations may be wide including hypothyroidism, T1D, hypergonadotropic hypogonadism, hypopituitarism and other autoimmune conditions. Hypoparathyroidism [89] and hypogonadism is more frequent in females than in males, while other endocrinopathies such as hypothyroidism do not seem to be influenced by sex. The gene responsible for APECED is AIRE, which in humans is selectively expressed in lymphoid tissues [90,91]. In the thymus, medullary epithelial cells (mTEC) express AIRE to promote antigen presentation and subsequent negative selection of autoreactive T cells that are generated during TCR rearrangement. Indeed, AIRE mutation causes a severe defect in the elimination of organ-specific autoreactive T cell clones, supporting the role of this gene in the regulation of central tolerance [92,93]. In addition, recent studies suggest an implication of AIRE deficiency in the development of B cell tolerance [94] leading to the production of a variety of autoantibodies directed against enzymes or intracellular proteins of the endocrine tissues and against cytokines [95]. Nearly 95% of APECED patients display anti-IFNα and anti-IFNω antibodies [96]. Anti-IFNωAbs were found to be the best serological marker of APECED regardless of the comorbidities and the duration of the syndrome. These antibodies can be particularly helpful in early diagnosis in patients presenting with a single typical manifestation [97]. Furthermore, antibodies directed against IL-17 and IL-22 have been identified, and these are linked to a disruption of Th17-mediated immunity that underlies the characteristic infectious vulnerability of this condition, chronic mucocutaneous candidiasis [98].

### 5.3. IEIs with Altered Peripheral Tolerance Mechanisms

#### 5.3.1. Immune Dysregulation, Polyendocrynopathy, Enteropathy, X-Linked

Among monogenic defects resulting in Treg cells dysfunction IPEX (Immune dysregulation, polyendocrynopathy, enteropathy, X-linked) syndrome represents the most relevant clinical condition caused by *FOXP3* gene alterations [99]. *FOXP3* gene encodes for a transcription factor which is crucial for Treg cells development in the thymus [100]. Treg are anergic lymphocytes, but upon activation they are able to suppress proliferation and IL-2 production of naive and memory T cells through a contact-dependent cytokine- independent mechanism. The absence of Treg cells has been associated with autoimmune diseases in both humans and mice [101]. Moreover, FOXP3 inhibits Th17 cell differentiation and IL-17 production. The inability to properly regulate Th17 response represents an additional mechanism leading to autoimmunity in IPEX syndrome [102]. The typical clinical phenotype of IPEX syndrome is the triad eczematous dermatitis, polyendocrynopathy [103], and enteropathy with intractable diarrhea. T1DM represents the most common endocrine manifestation observed in the majority of patients including newborns. The onset of IPEX syndrome usually occurs in males within their first few months of life and, if not promptly recognized, it can be fatal in early infancy. Therefore a timely diagnosis is essential to start appropriate treatment [104]. Patients with IPEX may also experience a wide range of organ-specific autoimmune conditions and severe viral, bacterial and fungal infectious diseases, such as airways and gastrointestinal infections and skin super-infections [105]. Thyroid autoimmunity is a common finding in IPEX patients, presenting either with hypothyroidism or, less frequently, with hyperthyroidism [106].

#### 5.3.2. CTLA-4 Haploinsufficiency

The protein cytotoxic T lymphocyte antigen-4 (CTLA-4) is a T coinhibitory molecule with the ability of extrinsically inhibit T cell proliferation and effector functions [105]. CTLA-4 is an essential effector component of Treg cells and is required for their suppressive function, contributing to the peripheral tolerance maintenance. Heterozygous germline mutations in CTLA-4 resulting in its haploinsufficiency or impaired ligand binding leads to a complex syndrome with features of both autoimmunity and immunodeficiency [107]. The most common clinical features of CTLA-4 haploinsufficiency include hypogammaglobulinemia, lymphocytic infiltration of nonlymphoid organs like CNS, lungs, GI tract, malignancies and B cells abnormalities which may account for antibody-mediated autoimmunity [108]. In a large worldwide cohort study published in 2018, the clinical spectrum of CTLA-4 deficiency have been examined; AITDs are reported as symptom/sign at the presentation in 5% of patients and globally autoimmune endocrinopathy affects 33% of patients [109].

#### 5.3.3. STAT3 Gain of Function

STAT3 is a transcription factor that mediate cellular response to cytokines and growth factors. STAT 3 gain-of-function (GOF) mutations were first described in 2014 to cause early-onset autoimmunity, lymphoproliferation and growth failure [110,111]. Early-onset immunodysregulation is paradigmatic in this IEI and AITD is one of the most common organ-specific autoimmune manifestation observed in about 30% of the cases [112]. STAT3 GOF causes not only increased STAT3-mediated transcriptional responses but also an alteration of the activity of other STATs. Moreover, STAT3 GOF restrains FoxP3 expression and Treg development [113].

#### 5.3.4. Autoimmune Lymphoproliferative Syndrome (ALPS)

Autoimmune lymphoproliferative syndrome (ALPS) is a rare condition characterized by lymphoproliferation and multiple autoimmunity. ALPS is due to defects in the intrinsic and extrinsic pathway of apoptosis. In particular, the most common mutation associated with ALPS is FAS mutation, described in 70% of cases [114]. Recently, mutations in other genes are being discovered in patients with ALPS-like phenotypes [115]. FAS is a member of the tumor necrosis factor receptor (TNFR) superfamily involved in caspases-mediated apoptosis. The role of this pathway on autoimmunity is crucial in maintaining lymphocyte homeostasis and peripheral immune tolerance. Moreover, TCRαβ+CD3+ CD4−CD8− cells, also known as double negative cells (DNTs), mature post-thymic T cells, not only represent an hallmark of ALPS, but also have a pathogenetic role, being involved in the production of autoantibodies [116]. AITD is a common autoimmune manifestation of ALPS. Recent studies highlighted the correlation between FAS -670A/G and -1377 G/A polymorphisms and the risk of developing AITD and other autoimmune disorders [117].

#### 5.3.5. STAT1 Gain of Function

STAT1 is mainly activated by the binding of type I interferons to their cellular receptors, being an effector of Interferon and Interferon signaling pathways. The cytokines receptor activation leads to the JAK-STAT molecules recruitment, subsequent STAT phosphorylation (pSTAT) and their translocation to the nucleus, where they regulate gene transcription [118]. STAT1 mutations increasing its phosphorylation, result in a gain of function effect for the upstream cytokines. STAT1 GOF mutations cause an autosomal dominant IEI characterized by early-onset chronic mucocutaneous candidiasis [119], susceptibility to mycobacterial, viral and fungal infections, multiorgan autoimmunity, vascular anomalies and malignancies [120]. Autoimmunity in STAT1 GOF seems to be related to inappropriate lymphocyte activation and signaling. Patients with STAT1 GOF show impaired natural-killer-cell function, abnormal T helper (Th) type 1 cells and follicular helper T-cell responses, and decreased type 17 helper T (Th17) polarization [121]. Moreover, autoantibodies are detectable in most patients, thus suggesting that abnormal STAT1 signaling in B cells might also account for the pathogenesis of autoimmunity [110]. A much higher rate of individuals displaying autoimmune manifestations (37%) has been described in a large court of STAT1 GOF patients compared to the standard population (3%). Most of these features were related to thyroid disease including hypothyroidism requiring hormone substitution and hyperthyroidism [122].

## 6. Therapeutic Strategies

Understanding the molecular basis of autoimmunity in IEIs enables the development and use of targeted strategies as potential tailored treatment. The therapeutic approach for autoimmune manifestations in IEIs ranges from traditional and nonspecific immunosuppressive drugs (e.g., glucocorticoids) to biological molecules targeting the specific immune pathway or molecular defect of the patient (Table 2). The latter strategy represents a dramatically fast evolving field in IEIs, with the aim of controlling the disease manifestations with the minimum side effects [123,124]. Anti-TNF agents are a consolidated strategy to control autoimmune and autoinflammatory manifestations in patients with CVID, STAT3 GOF and others [125]. This group of molecules includes Infliximab, Adalimumab and Etanercept, which have been demonstrated to be best effective for the treatment of gastrointestinal and rheumatological symptoms [126]. Among other agents targeting inflammatory cytokines, Tocilizumab is a monoclonal antibody inhibiting the transduction pathway downstream IL-6. Since it was initially introduced for the treatment of rheumatoid arthritis, currently it’s been demonstrated to be effective in patients with STAT3 GOF with immune dysregulatory diseases, not controlled by traditional immunosuppressive drugs [127]. For the treatment of immune dysregulatory manifestations in patients with IEIs affecting JAK-STAT pathway, JAK Inhibitors have been developed due to their effect on inflammatory cytokines synthesis reduction. Since both STAT1 and STAT3 GOF have significant morbidity and mortality, nowadays JAK inhibitors constitute an effective treatment for severe organ specific autoimmunity in patients, monitoring the risk of infectious side effects [128,129]. Tofacitinib is the first molecule to have been used in humans targeting JAK1 and JAK3; both Baricitinib and Ruxolitinib targets JAK1 and JAK2 [127]. These targeted molecules directly acting on the hyperactivated JAK-STAT pathway have shown a potential role in reverting the underlying mechanisms leading to organ specific autoimmunity. The preliminary effects have been described in STAT1 GOF-related DM1 [121]. Abatacept is a fusion protein CTLA4-Ig which constitutes a current therapeutic option for patients with CTLA-4 deficiency. Its efficacy in the control of severe autoimmunity has been mainly demonstrated for autoimmune enteropathy, partially restoring T reg activity which is defective in these patients [130].

First approved for the treatment of hematological malignancies, B cell targeted therapies currently play a major rule as therapeutic strategy for autoimmune manifestations associated with IEIs. The use of Rituximab, a monoclonal antibody anti CD20, has the effect of B cell depletion which is exploited to control autoimmunity like autoimmune cytopenia associated with CVID [79]. Despite CD20 is not expressed of hematopoietic stem cells (HSC), some patients treated with Rituximab may develop permanent B cell deficiency and hypogammaglobulinemia requiring Ig replacement therapy as side effect [131].

To date, evidence regarding the beneficial effects of targeted therapies on AITD remains scarce. Although the impact of JAK inhibitors on AITDs in patients with *STAT1* and *STAT3* GOF variants has not yet been specifically investigated, JAK inhibitors, including ruxolitinib, have demonstrated efficacy in the control of autoimmune manifestations across different clinical settings [132]. Improvement in T1DM has been described, with isolated reports of complete disease remission and discontinuation of insulin therapy [121]. Similarly, a recent report has suggested that ruxolitinib treatment in patients with APECED may also affect thyroid function, with normalization of thyroid parameters and discontinuation of replacement therapy observed in a treated patient. These data are in line with the observation that immunomodulatory strategies may represent a potential therapeutic option for the management of selected autoimmune manifestations, even in the absence of a defined IEI [129,133,134]. However, considerations on the risk/benefit balance are mandatory for every single condition. In fact, despite an overall favorable safety profile reported to date, the use of JAK inhibitors requires careful infectious and oncological surveillance, given their immunomodulatory effects. Furthermore, as these agents have been introduced into clinical practice relatively recently, prospective studies are needed to better characterize their long-term safety and efficacy. Studies are also needed to define the effect of these treatments on the progression of autoimmune manifestations in patients with multiple autoimmunity. In this direction, future research should aim at identifying predictors of autoimmunity spreading to other organs [128,132,135]. For example, the presence of clinical “red flags,” such as resistance to standard therapies, early disease onset, or mixed phenotypes combining autoimmunity with recurrent or severe infections should raise the suspicion of an underlying genetic defect consistent with an IEI and prompt targeted treatment [69,136].

## 7. Conclusions

In recent years, immune dysregulation has emerged as a central hallmark of inborn errors of immunity, offering a unique window into the mechanisms that drive autoimmunity. The molecular pathways disrupted in these conditions provide valuable models for dissecting the complex interplay between genetic, epigenetic, and environmental factors including EDCs, as well as the contribution of distinct immune compartments. Notably, AITDs represent a frequent clinical manifestation across several IEIs, often emerging as part of a broader immunodysregulatory phenotype. Although isolated AITDs are typically polygenic and multifactorial, it is plausible that the discrete mechanisms of immune tolerance breakdown identified in monogenic IEIs, such as defects in central tolerance, regulatory T-cell dysfunction, or aberrant cytokine signaling, may also contribute to the pathogenesis of common isolated AITDs. This conceptual overlap reinforces the translational relevance of IEIs as mechanistic models, offering valuable insights into the immunological checkpoints that may be subtly disrupted in polygenic autoimmunity.

Insights gained from these rare disorders significantly enhance our understanding of the pathogenic processes underlying common autoimmune diseases such as autoimmune thyroiditis, and may ultimately contribute to refining diagnostic and therapeutic approaches.

## Figures and Tables

**Table 1:** Selected IEIs associated with AITDs.

Disease	Gene	Main Clinical Features
Selective IgA deficiency	—	Recurrent infections, allergic diseases, autoimmune manifestations including AITDs
CVID	Multiple (e.g., *TNFRSF13B*, *NFKB1*)	Hypogammaglobulinemia, recurrent infections, autoimmunity
APECED	*AIRE*	Chronic mucocutaneous candidiasis, poliendocrinopathy, hypoparathyroidism, adrenal insufficiency ectodermal distrophy;
IPEX	*FOXP3*	Enteropathy, type 1 diabetes, eczema, autoimmune endocrinopathies
CTLA4 haploinsufficiency	*CTLA4*	Hypogammaglobulinemia, lymphoproliferation, malignancies, autoimmunity
STAT3 GOF	*STAT3*	Early-onset autoimmunity, growth failure, interstitial lung disease
STAT1 GOF	*STAT1*	Chronic mucocutaneous candidiasis, autoimmunity, susceptibility to fungal and viral infections

**Table 2:** Immune checkpoint categories and targeted drugs in IEIs with thyroid autoimmunity.

Disease	Immune Checkpoint	Drug
CVID	B-cell tolerance	Rituximab
APECED	Central tolerance	Rituximab Ruxolitinib
IPEX	Regulatory compartment	
CHAI	Regulatory compartment	Abatacept
STAT3 GOF	Regulatory compartment/ JAK-STAT pathway	Tocilizumab Ruxolitinib
STAT1 GOF	Increased IFNI signature/ JAK-STAT pathway	Ruxolitinib Tofacitinib

## Data Availability

No new data were created or analyzed in this study. Data sharing is not applicable to this article.

## Abbreviations

The following abbreviations are used in this manuscript:

ACE2	Angiotensin-Converting Enzyme 2
AITD/AITDs	Autoimmune Thyroid Disease(s)
ALPS	Autoimmune Lymphoproliferative Syndrome
APECED	Autoimmune Polyendocrinopathy-Candidiasis-Ectodermal Dystrophy
APS1	Autoimmune Polyendocrine Syndrome type 1
BAFF	B-cell Activating Factor
BCR	B-cell Receptor (implicit in context)
CD	Celiac Disease
CMC	Chronic Mucocutaneous Candidiasis
CTLA-4	Cytotoxic T-Lymphocyte Antigen 4
CV	Cardiovascular
CVID	Common Variable Immunodeficiency
DM1/T1DM	Type 1 Diabetes Mellitus
DNTs	Double-Negative T cells (TCRαβ+CD3+CD4−CD8−)
EDCs	Endocrine-disrupting chemicals
GI	Gastrointestinal
HT	Hashimoto Thyroiditis
HSC	Hematopoietic Stem Cells
IEI/IEIs	Inborn Error(s) of Immunity
IFN	Interferon
IPEX	Immune Dysregulation, Polyendocrinopathy, Enteropathy, X-linked
mTEC	Medullary Thymic Epithelial Cells
miRNA/miR	MicroRNA
PFAS	polyfluoroalkyl substances
pSTAT	Phosphorylated STAT
PS1	Polyendocrine Syndrome type 1 (synonym of APS1)
SIgAD	Selective IgA Deficiency
SNP	Single Nucleotide Polymorphism
TACI	Transmembrane Activator and CAML Interactor
TG/Tg-Ab	Thyroglobulin/Anti-Thyroglobulin Antibodies
THSR/TSHR	Thyroid-Stimulating Hormone Receptor
TPO/TPO-Ab	Thyroid Peroxidase/Anti-Thyroid Peroxidase Antibodies
TRAb	TSH Receptor Antibodies
Treg	Regulatory T cells
